# Role of Lamin A/C Gene Mutations in the Signaling Defects Leading to Cardiomyopathies

**DOI:** 10.3389/fphys.2018.01356

**Published:** 2018-09-25

**Authors:** Andrea Gerbino, Giuseppe Procino, Maria Svelto, Monica Carmosino

**Affiliations:** ^1^Department of Biosciences, Biotechnologies and Biopharmaceutics, University of Bari Aldo Moro, Bari, Italy; ^2^Department of Sciences, University of Basilicata, Potenza, Italy

**Keywords:** nucleus, lamin A/C gene, nuclear lamina, nuclear envelope, gene expression, signaling pathways, Ca^2+^ signaling, cardiac pathophysiology

## Abstract

Nuclear lamin A/C are crucial components of the intricate protein mesh that underlies the inner nuclear membrane and confers mainly nuclear and cytosolic rigidity. However, throughout the years a number of other key physiological processes have been associated with lamins such as modulation of both genes expression and the activity of signaling mediators. To further solidify its importance in cell physiology, mutations in the lamin A/C gene (*LMNA*) have been associated to diverse pathological phenotypes with skeletal muscles and the heart being the most affected systems. When affected, the heart develops a wide array of phenotypes spanning from dilated cardiomyopathy with conduction defects to arrhythmogenic right ventricular cardiomyopathy. The surprising large number of cardiac phenotypes reflects the equally large number of specific mutations identified in the *LMNA* gene. In this review, we underlie how mutations in *LMNA* can impact the activity and the spatial/temporal organization of signaling mediators and transcription factors. We analyzed the ever-increasing amount of findings collected in Lmna^H222P/H222P^ mice whose cardiomyopathy resemble the most important features of the disease in humans and a number of key evidences from other experimental models. With this mini review, we attempt to combine the newest insights regarding both the pathogenic effects of *LMNA* mutations in terms of signaling abnormalities and cardiac laminopathies.

## Introduction

A-type nuclear lamins are 3.5 nm diameter type V intermediate filament proteins expressed in the majority of differentiated mammalian somatic cells, including cardiomyocytes (Butin-Israeli et al., 2012). Lamin A and C are encoded by the same gene (*LMNA*, cytogenetic location: 1q22) thanks to alternative RNA splicing events (Lin and Worman, 1993) and targeted to the nucleus by a nuclear localization signal (NLS). Both proteins polymerize with B-type lamins to form the nuclear lamina (NL) a scaffold apposed to the inner nuclear membrane (INM) of metazoan cells. For more information regarding cellular and tissue expression of all lamin isoforms, their structure and assembly readers are referred to these reviews (Ho and Lammerding, 2012; Schreiber and Kennedy, 2013). Recently, Turgay et al. (2017), used cryo-electron tomography to gain insight about the architecture of the thin lamin meshwork within the NL. This mesh of proteins confers cellular and nuclear integrity against mechanical cues. Additionally, thanks to its interaction with over 100 cytosolic and intra nuclear membrane/nucleoplasmic proteins(Gerace and Tapia, 2018), the NL allows a wide number of other functions such as mechano-transduction, chromatin protection/organization, regulation of signaling, and gene expression (Carmosino et al., 2014; Swift and Discher, 2014; Gerace and Tapia, 2018). The unexpected involvement of the NL in a large array of physiological processes triggered the scientific interest and these proteins became the focus of a multidisciplinary field.

Lamin A/C gene is among the most common cardiomyopathy-causing gene (van Tintelen et al., 2007a). Hundreds of distinct *LMNA* mutations have been associated with 15 heritable and organ-specific or multisystem disease phenotypes such as accelerated aging or overlapping syndromes (Cattin et al., 2013b) which are usually identified as laminopathies (Scharner et al., 2018). Mostly, these diseases affect specifically the striated muscle with a recurrent involvement of the heart that develops different type of arrhythmogenic cardiomyopathies with high interfamilial heterogeneity being dilated cardiomyopathy with conduction defects (DCM-CD) the most prevalent. However, over the years, *LMNA* mutations have been also associated with a combination of morpho-functional phenotypes between DCM and arrhythmogenic right ventricular cardiomyopathy (ARVC) (Forleo et al., 2015), underlining the urge for a new (and wider) classification of cardiac laminopathies (Arbustini et al., 2013). Strikingly, in all the cardiac phenotypes reported, structural abnormalities and electrical instability always coexist being myocardial fibrosis a key player in the development of both types of cardiac impairment.

Cardiomyopathies induced by mutations in *LMNA* have a very aggressive and fast clinical course that could culminate with sudden cardiac death from malignant ventricular arrhythmias and end-stage heart failure occurring at earlier ages compared to other familial cardiomyopathies. Current therapies involve implantable pacemakers and defibrillators to manage arrhythmia and conduction defects in order to prevent sudden cardiac death (Peretto et al., 2018). The pharmacological interventions commonly focus on the symptoms of congestive heart failure. These therapies extend the survival rate of affected patients; however, they only improve cardiac function and decrease the complications and secondary features of the disease without focusing on the specific mutation causing the pathology. Unfortunately, the design of patient-specific therapeutic approaches is often hindered by the lack of insight regarding the underlying pathogenic mechanisms.

Two not competing hypothesis could explain the etiology of laminopathies. The *mechanical stress* hypothesis proposes that the reduced nuclear rigidity due to Lamin A/C mutations could increase cellular susceptibility against recurrent mechanical stress and reduce mechano-transduction, especially in cells subjected to mechanical forces such those of skeletal muscle and cardiomyocytes (Carmosino et al., 2014). On the other hand, the *gene expression* hypothesis proposes that mutation-induced defects in proteins of the nuclear envelope might lead to changes in signaling pathways and abnormal control of gene expression which, in turn, could be associated to skeletal muscle diseases (Chatzifrangkeskou et al., 2015) and cardiomyopathies (Worman, 2017).

Here we describe the newest insights regarding the mechanisms by which *LMNA* mutations impact diverse cardiac signaling pathways and intracellular mediators in an updated brief review.

## One Mutation, Multiple Phenotypic Features

**FIGURE 1:**
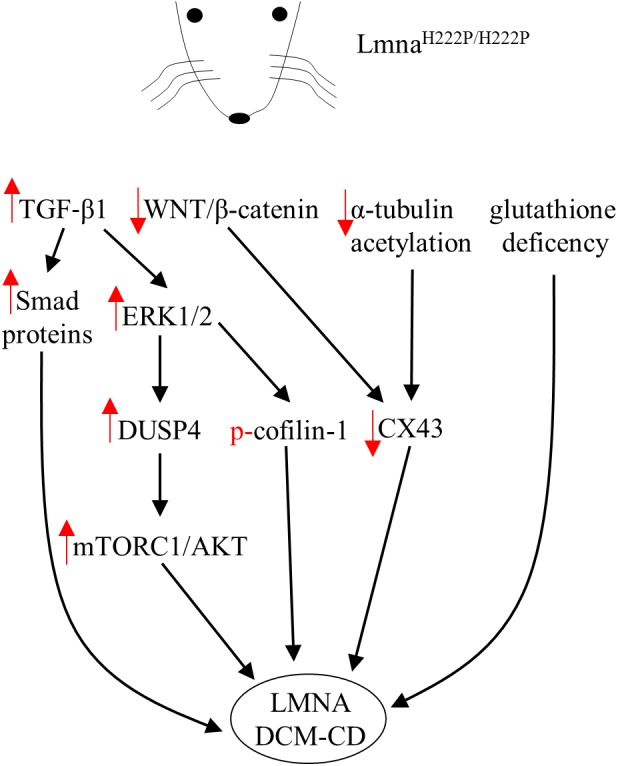
Diagram showing signaling mediators and cellular targets affected in hearts of Lmna^H222P/H222P^ mice. Red arrows indicate the effects (increase/decrease) induced by the mutation in terms of expression level or activity.

Specific *LMNA* mutations can induce alterations in the interplay between lamins themselves or their interactors of the NL and core signaling factors potentially involved in transcriptional regulation in both cells and animal tissues (Worman, 2017). This may trigger diverse pathophysiological mechanisms that underpin the broad spectrum of clinical phenotypes described in the above paragraph. The lack of cardiac tissue samples from affected patients has limited our ability to identify the precise molecular mechanisms through which each lamin mutation functions, however, the generation of the first transgenic lamin A/C knockout mouse paved the road for overcoming this limitation (Sullivan et al., 1999). Since then, different transgenic mice, baring specific *LMNA* mutations, have been generated and reviewed in Brayson and Shanahan (2017). For instance, most of the evidences regarding all the signaling pathways and the specific targets modulated in the striated muscle by a single *LMNA* mutation rely on a single mouse model containing a knock-in mutation in lamin A/C gene (H222P, **Figure 1**) causing muscular dystrophy and DCM (Arimura et al., 2005). Lmna^H222P/H222P^ mice recapitulate some of the most important features of cardiac laminopathies in humans such as left ventricular dilation and systolic dysfunction with male mice developing the disease with faster kinetics relative to female mice (Choi and Worman, 2014; Worman, 2017).

### ERK1/2 Signaling

Lamins are well-known molecular scaffolds that tether at the nuclear envelope mitogen-activated protein (MAP) kinase extracellular signal-regulated kinase (ERK) 1/2 and its downstream transcription factor c-Fos, a major regulator of important cellular processes such as cell proliferation, death, survival, and differentiation (Shaulian and Karin, 2002; Eferl and Wagner, 2003). Mutation-induced alterations in lamin A/C structure and or function might perturb the nuclear envelope geometry sufficiently to directly affect ERK1/2 activity and consequently its downstream signaling targets. In fact, ERK1/2 enhanced phosphorylation and nuclear sequestration were reported in the heart of patients with cardiolaminopathy (Muchir et al., 2007). In the hearts of Lmna^H222P/H222P^ mice, hyper-activation of ERK1/2, JNK and P38α was reported before the onset of significant cardiac impairments (Muchir et al., 2007, 2012a) likely indicating that MAP kinases hyperactivation is not a consequence but rather a causative event of the cardiac disease. Strikingly, genetic or pharmacological approaches used to control ERK1/2 signaling in Lmna^H222P/H222P^ mice had a positive outcome on cardiac symptoms led to survival prolongation (Muchir et al., 2012a; Wu et al., 2011, 2014, 2017). In addition, several downstream target genes were activated by MAPKs in hearts of Lmna^H222P/H222P^ mice (Wu et al., 2011, 2017; Muchir et al., 2012a; Chatzifrangkeskou et al., 2016). All these targets might in turn, modulate the expression of additional genes encoding for proteins potentially involved in pathogenic mechanisms of cardiac diseases (Gillespie-Brown et al., 1995; Thorburn et al., 1995). The mechanisms by which expression of Lmna^H222P^ leads to ERK1/2 signaling hyperactivation are under investigation. However, in the heart of Lmna^H222P/H222P^ mice, ERK1/2 activation was functionally preceded by the elevation of both the transforming growth factor beta-1 (TGF-β1), an important mediator of fibrosis and extracellular matrix deposition (Rosenkranz, 2004), and its nuclear effectors, Smad proteins (Chatzifrangkeskou et al., 2016). Of note, treatment with a TGF-β receptor blocker lowered the amount of activated ERK1/2, indicating an increased TGF-β1 upstream ERK1/2 activation, as already reported for other cardiac pathophysiological states (Huang et al., 2015).

Recently, Chatzifrangkeskou et al. (2018) demonstrated that active cytosolic ERK1/2 can directly interact and phosphorylate cofilin-1, an F-actin depolymerizing factor. When phosphorylated, cofilin-1 impacts actin dynamics, which in turn leads defective actin organization in sarcomeres and alterations in contractile force generation of the left ventricular. Of note, left ventricular tissue from wild type mice injected with adeno-associated viruses expressing cofilin-1 showed myofibrillar disruption similar to that observed in Lmna^H222P/H222P^ mice. Thus, pharmacological compounds able to correct defective actin dynamics could be used as new therapeutic approach to improve left ventricular dysfunction in *LMNA*-mediated cardiomyopathy (Chatzifrangkeskou et al., 2018).

### AKT/mTORC1 Signaling

One of the direct consequences of ERK1/2 hyperactivation and nuclear sequestration is the increased expression of the dual specific protein phosphatase 4 (DUSP4) together with left ventricular dilation markers in ventricular tissue of Lmna^H222P/H222P^ (Choi et al., 2012b, 2018). DUSP4 is an ERK1/2-specific phosphatase predominantly located in the nucleus where it is involved in regulating the ERK1/2 cascade at nuclear level. Of note, genetic deletion of DUSP4 ameliorated heart function and prolonged survival in Lmna^H222P/H222P^ mice (Choi et al., 2018). So far, this phosphatase is the leading molecular link between MAP kinases and the hyperactivation of protein kinase B/mammalian target of rapamycin complex 1 (mTORC1) signaling pathway in Lmna^H222P/H222P^ mice (Choi et al., 2012b) and likely related to reduced autophagy, a key process for lifespan extension (Eisenberg et al., 2009; Morselli et al., 2010). Strikingly, treatment with temsirolimus, a specific inhibitor of mTOR, restores autophagy and prevents cardiac defects in Lmna^H22P/H22P^ mice (Choi et al., 2012a).

### WNT/β-Catenin Signaling

Furthermore, Lmna^H222P/H222P^ mice also showed reduced expression of WNT and β-catenin (Muchir et al., 2007, 2012b; Le Dour et al., 2017), two signaling mediators involved in several cellular mechanisms leading to proliferation, differentiation and apoptosis. β-catenin is usually phosphorylated, in the absence of WNT stimulation, by GSK-3β and degraded after ubiquitination by the proteasome. However, upon WNT activation, GSK-3β is inactivated and β-catenin firstly accumulates in the cytosol and then translocates to the nucleus where it forms a complex with transcription factors, which in turn activate target genes. In the heart β-catenin can interact with the gene encoding connexin 43 (CX43), an important cardiac connexin, to increase its transcription. In addition, β-catenin can also interact with CX43 itself, as part of a multiproteic complex within the intercalated disk, to stabilize its structure (Le Dour et al., 2017). Thus, as shown in Lmna^H222P/H222P^ mice, the reduced CX43 expression seems tightly dependent by the deficient WNT/β-catenin signaling, since the pharmacological rescue of β-catenin obtained by blocking its specific inhibitor GSK-3β, restores the levels of CX43. Accordingly, mice lacking Lamin A have also decreased CX43 levels, while mutant mice homozygous for the Lmna N195K variation have similar conduction abnormalities induced by altered expression and distributions of both CX40 and CX43 and misregulation of HF1b/Sp4 a transcription factor of the Sp family (Mounkes et al., 2005). In addition, CX43 protein expression was reduced by about 40% in neonatal rat cardiomyocytes expressing Lmna E82K (Chen et al., 2013). Furthermore, alterations of the cytoskeleton through reduced acetylation of α-tubulin have to be taken into account when considering the abnormal distribution of CX43 in cardiomyocytes and cardiac tissue from Lmna^H222P/H222P^ mice. Treatment with paclitaxel, in fact, stabilized the microtubule network via increased acetylation of α-tubulin and this, in turn, helped the correct localization of CX43 at intercalated disks improving conduction defects in Lmna^H222P/H222P^ hearts (Macquart et al., 2018).

Finally, Rodriguez et al. (2018) showed that the cardiac phenotype associated with Lmna^H222P/H222P^ underlines abnormal oxidative stress levels and glutathione deficiency. Of note, pharmacological glutathione replenishment lowered cardiac oxidative stress damage and mitigates contractile dysfunction in Lmna^H222P/H222P^ mice (Rodriguez et al., 2018).

All the findings collected using Lmna^H222P/H222P^ mice unveiled the unexpected impact that a single mutation of a structural gene may have in cell signaling. This animal model made us realize of the urge for the generation of new transgenic models in order to evaluate the impact of other mutations of lamin A/C. Hopefully, the collected information will help us discover the molecular mechanisms that underpin the surprising functional complexity of this protein.

## Apoptosis, Endoplasmic Reticulum-Stress and the Ca^2+^ Signaling

Additional insights on the pathogenic mechanisms induced by *LMNA* mutations have been obtained using other experimental models. Specific *LMNA* mutations can significantly impact the dynamic equilibrium between cell death and survival in response to mechanical or metabolic cues. Cell death through apoptosis causes continuous loss of myocytes and leads to alterations of cardiac conduction and dispersion of refractoriness (Sen-Chowdhry and McKenna, 2012), potentially resulting in sudden cardiac death (James, 1994). In addition, reduction in cardiomyocytes content, likely compensated by additional fibrosis, may represent a substrate for conduction block and re-entrant arrhythmias (Basso et al., 2012; Sen-Chowdhry and McKenna, 2012). Indeed, previous reports have shown that *LMNA* mutation carriers with conduction defects and arrhythmias have histopathological signs of myocardial fibrosis that involves the cardiac conduction system (Fatkin et al., 1999; Arbustini et al., 2002; van Tintelen et al., 2007b).

Indeed, Lmna^E82K/E82K^ transgenic mice were affected by DCM-CD, myocyte disarray and collagen accumulation. Lmna E82K was mislocalized in the nucleus with impaired nuclear envelope integrity, swollen mitochondria with reduced number of cristae. Of note, expression of Lmna E82K increased 9 fold the apoptosis rate, probably as consequence of first apoptosis signal receptor (FAS) overexpression, with activation of caspase-8/caspase-3 and caspase-9-dependent release of cytochrome c from mitochondria (Lu et al., 2010).

**FIGURE 2:**
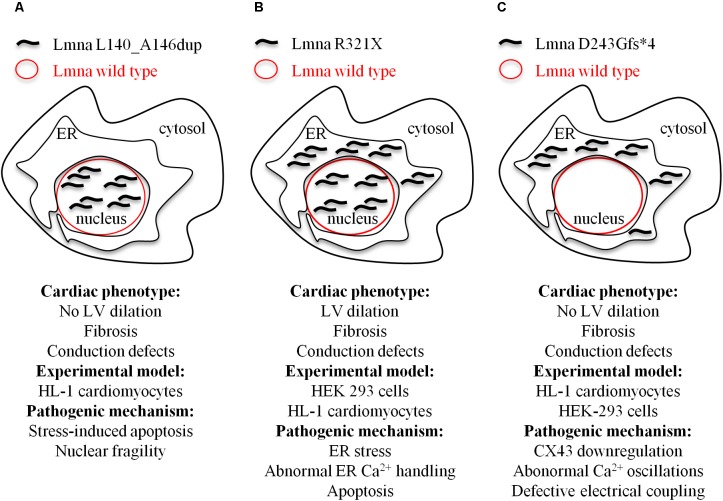
Model depicting the cellular mislocalization of the three mutants of lamin A in light of the cardiac phenotypes of the probands and the experimental models used for the evaluation of the pathogenic mechanisms at cellular level. **(A)** Lmna L140_A146dup; **(B)** Lmna R321X; and **(C)** D243Gfs^∗^4.

We identified in members of an Italian family a novel heterozygous lamin A/C variant consisting of a in-frame duplication of 21 nucleotides in the exon 2 of the *LMNA* gene (Forleo et al., 2015). This mutation co-segregates with arrhythmogenic cardiomyopathy of different phenotypes with high intra-familial variability. The *in vitro* characterization of this new *LMNA* variant showed a decreased nuclear stability and impaired nuclear-cytoskeletal coupling, resulting in a higher susceptibility to nuclear rupture and stress-induced cardiomyocyte apoptosis as the main pathogenic mechanism (**Figure 2A**).

Cardiolaminopathies are not only the result of missense mutations. Mutations like nonsense or splice-site or insertions/deletions are more rare (van Rijsingen et al., 2012; Kawakami et al., 2018) but result in more severe cardiac events. We characterized at cellular level two different nonsense mutations, Lmna R321X (Carmosino et al., 2016) and Lmna D243Gfs^∗^4 (Gerbino et al., 2017), both segregating with cardiomyopathies with conduction defects (**Figures 2B,C**). Both mutations generated a premature termination codon before the NLS. When exogenously expressed in HEK293 or HL-1 cardiomyocytes both mutants mislocalized and accumulated within the endoplasmic reticulum (ER). Of note, Lmna R321X ER-accumulation led to unfolded protein response (UPR), an adaptive mechanism involving a complex transcriptional program that induces the hyperphosphorylation of PERK, a kinase localized on the ER membrane (Oslowski and Urano, 2011). PERK phosphorylates eIF2 leading to the expression of transcription factors such as CHOP, ultimately promoting the expression of proapoptotic genes. In our study, we found increased expression of both p-PERK and CHOP in cells expressing Lmna R321X and increased levels of p-PERK in samples of the heart explanted from a mutation carrier. If not compensated, UPR leads to apoptosis since the cellular expression of Lmna R321X itself was able to significantly increase the apoptotic rate when compared with cells expressing the wild type Lamin A. Thus, the fibro-fatty replacement of the cardiac tissue could well explain the conduction defect diagnosed in patients carrying this specific mutation (Carmosino et al., 2016). An alternative pathogenic mechanism that leads to DCM in Lmna^ΔK32/+^ mice was showed by Cattin et al. (2013a). These authors proposed a scenario in which haploinsufficiency induced by ΔK32 degradation caused cardiac remodeling which, in turn, impaired UPR. Consequently, cardiac function worsened leading to DCM due to toxic accumulation of both lamin wild type and ΔK32 (Cattin et al., 2013a).

When we functionally characterized the second nonsense *LMNA* mutation, we found that ER Lmna D243Gfs^∗^4 accumulation did not induce neither UPR/ER stress nor apoptosis (Gerbino et al., 2017). The fact that the Lmna D243Gfs^∗^4 mutant was silent in terms of UPR suggested its aggregation in a more tolerated unfolded state likely because of the lack of the whole coil 2b region, which is involved in the polymeric head-to-coil assembly of Lamin monomers (Strelkov et al., 2004). While performing experiments to evaluate the impact of ER Lmna D243Gfs^∗^4 accumulation on intracellular Ca^2+^ dynamics we measured a significant impairment of Ca^2+^ oscillations both in the cardiomyocyte carrying the mutation and in the cardiomyocyte directly coupled with it, indicating defective electrical coupling between these cardiac cells. Under this scenario, we showed that HL-1 cardiomyocytes expressing Lmna D243Gfs^∗^4 had a significant reduction in CX43 expression level that was, however, independent of both β-catenin expression level and cytoskeleton polymerization (measured as RhoA activity). Of note, partial restoration of CX43 activity in Lmna D243Gfs^∗^4-expressing cells by lithium improved cell-to-cell signal propagation suggesting CX43 as potential pharmaceutical target for this form of cardiolaminopathy.

## Conclusion and Future Perspectives

During the functional characterization of both truncated mutants we uncovered critical changes in intracellular Ca^2+^ dynamics. On one hand, Lmna R321X ER accumulation led to an abnormal Ca^2+^ handling by the cell; ER Ca^2+^ content was significantly reduced as well as the Ca^2+^-mediated agonist responses in both the cytosol and the nucleus. On the other hand, Lmna D243Gfs^∗^4 expression affected the regular Ca^2+^ oscillatory pattern of cardiomyocytes through a CX-43 dysfunction. Thus, the effect of *LMNA* mutations on Ca^2+^ changes might represent a new and under-investigated event in the pathogenesis of *LMNA*-mediated cardiomyopathies. Of note, Arimura et al. (2010) showed that SCH00013, a Ca^2+^ sensitizer able to increase muscular Ca^2+^ sensitivity without increasing cytosolic Ca^2+^ levels, ameliorated systolic dysfunction, reduced cardiac interstitial fibrosis and modulated the expression of genes involved in cardiac remodeling when administrated in Lmna^H222P/H222P^ mice. Nuclear and cytosolic Ca^2+^ changes can activate Ca^2+^-dependent PKC isoforms either resident within the nucleus or translocated from the cytosol, respectively. Interestingly, lamin A has at least two consensus sites for PKC and others for kinases that can be modulated by Ca^2+^ (Machowska et al., 2015).

Overall, we believe that the exact dissection of all the cellular pathogenic mechanisms induced by mutations in lamin A/C gene will pave the road toward personalized treatment for these cardiomyopathies. Patients carrying different mutations with the same cardiac phenotype should not be therapeutically approached with the same strategy. For instance, cardiac fibrosis is not only induced by apoptosis but also by abnormal CX43 expression triggers due to enhanced activity of non-excitable cardiac fibroblasts (Jansen et al., 2012). Of note, normalization of CX43 expression might prevent fibrosis and reduce the susceptibility to fatal arrhythmia. Thus, a complete palette of all the pathogenic mechanisms involved in *LMNA*-mediated cardiomyopathies will help cardiologists to design more precise and effective therapeutic protocols.

## Author Contributions

AG conceived the idea of this article. AG and MC wrote and edited the manuscript. Both GP and MS reviewed and critically edited the content of the manuscript.

## Conflict of Interest Statement

The authors declare that the research was conducted in the absence of any commercial or financial relationships that could be construed as a potential conflict of interest. The reviewer DB and handling Editor declared their shared affiliation at the time of the review.
